# Community-based organizations are critical partners in providing complete cancer care

**DOI:** 10.3747/co.v16i2.357

**Published:** 2009-03

**Authors:** R. Rutledge, L. Robinson

**Keywords:** Diagnosis, support, survivorship

## Abstract

In Canada people affected by cancer access psychosocial care and support from two systems. In the conventional medical system, psychosocial professionals focus mainly on screening for and treating those most distressed by their diagnosis. Many patients and family members go beyond this step to find information and support provided by community-based organizations (cbos). This article outlines the components of complete cancer care effectively provided by cbos and why the integration of the two systems of care is critical in delivering seamless high-quality psychosocial care for all.

## 1. INTRODUCTION

Since the end of the 1990s, Canada has witnessed the successful development of dozens of organizations dedicated wholly or in part to fulfilling the psychosocial needs of people affected by cancer. These community-based organizations (cbos) were created to fill the perceived gap in whole-person care currently not filled by the medical system. The purpose of the present article is to provide an overview of how the cbos currently contribute to complete cancer care, and to outline a vision of how a true partnership between the cbos and the medical system could better serve the growing number of cancer survivors.

## 2. BACKGROUND AND METHODOLOGY

In 2006, the board of the Canadian Association of Psychosocial Oncology, a group representing professional psychosocial specialists, invited leaders from 11 cbos to an exploratory meeting regarding the development of partnerships in psychosocial cancer care (Table I). The perception leading up to this meeting was that patients and families often seek psychosocial support from the medical system or the cbos (or both), but that little communication and coordination of services occurs between these two providers. The consensus from the meeting was that a need existed to develop a mechanism to share information such as program availability, psychosocial research findings, and best practices, and to develop common standards for educational materials. A vision arose of providing seamless high-quality psychosocial oncology care by developing a partnership of the medical system, psychosocial professionals, and cbos through knowledge exchange and capacity building—informed by psychosocial research, where appropriate.

In 2007, through grants provided by the National Cancer Institute of Canada and the Canadian Institutes of Health Research, a workshop titled “Creating a Community for Knowledge Exchange and Capacity Building in Psychosocial Cancer Care” was organized. The workshop was later named Canadian Psychosocial Oncology Partners (cpop) by the workshop participants. In preparation for the workshop, an environmental scan of the knowledge exchange practices of the cbos took place. Of the 14 Canadian cbos who provide psychosocial oncology services and who were approached for the workshop, 13 agreed to a taped interview.

The results of those interviews underwent thematic analysis. In brief, the results showed that

- cbos strongly endorse a system to exchange knowledge in psychosocial oncology;
- a central, online database was felt to be key to sharing information (although personal exchanges were felt to be very helpful as well); and
- the content of the information to be shared should include all aspects of service delivery, ways to efficiently share information with target audiences, and application of research to practice.

The inaugural workshop in May 2007 was attended by 10 health care professionals, 5 health researchers, and 16 representatives from cbos. The intent was to develop a community of practice in psychosocial oncology in which individuals with this common interest share their expertise. The primary task identified by the workshop attendees was to develop an online portal or clearinghouse to facilitate development of the community so as to enhance capacity for psychosocial oncology knowledge exchange.

The second cpop workshop in May 2008 involved 30 people representing the cbos, the medical system, researchers, and psychosocial professionals. This workshop further developed the community of practice and was charged with the task of developing an online mechanism to further the group’s goals.

The knowledge gained from the 2007 interviews with cbo leaders provides the basis for the opinions provided in the section on complete cancer care that follows.

## 3. DISCUSSION

### 3.1 How Do CBOs Contribute to the Delivery of Complete Cancer Care?

Table II lists the essential components of complete care. This list is neither completely comprehensive, nor likely to be taken up in its entirety by patients and their families. The vision is that every person affected by cancer, whether it be the person receiving a diagnosis or a family member or friend, should have access to the types of programming listed, and that we, with the larger community (inside and outside the medical system), should help guide people toward the programming and services most suited to their needs.

#### 3.1.1 Information

Helping people understand their cancer experience is imperative. The topics covered vary from the definition of cancer, its treatment, and how the medical system functions, to the role and responsibilities of patients with regard to their own health and health care delivery, and every other of element of complete care listed here. Understanding what is happening reduces anxiety, improves the quality of interactions with the medical system, and critically assists in the preparation for every visit with a professional team member^1^.

Ultimately, medical professionals are in the best position to help patients and families make sense of their situation, to inform patients about treatment options, and to further tailor the types of information they believe will be most helpful for each individual. All of the cbos interviewed were emphatic: they do not provide medical advice, but are able to help facilitate the process of information-seeking. Almost all of the information provided by cbos comes from the medical system. Through one-to-one contact with patients at every visit, the cbos ensure access by the patients to print materials, medical-system Web sites, library resources, additional public information sessions, and patient navigators, among other knowledge translation strategies. However, cbos are also in a unique position to empower people by imparting knowledge in ways not offered by the medical system:

- Patients often want to gain more information during non-working hours. Many cbos offer toll-free numbers available into evening hours and on weekends. Furthermore, some cbos offer access to information specialists in multiple languages.
- Often acting as knowledge brokers, cbos can arrange for information to be packaged and provided in a medium customized to the individual’s learning needs—for example, by sending patients electronic documents or print materials. Also, cbos can direct people to medical information services such as hospital libraries provided by the medical system.
- When cbos are highly motivated to help with the prevention and early detection of cancers (through promotion of screening programs), they can promote public health messages. They can raise awareness of health promotion and cancer prevention in ways that far exceed the results that the medical system can accomplish alone.

One concern of the medical system is that cbos may be providing outdated or invalid information. Critics point to the absence of national standards for educational materials and the inability of the lay public to easily evaluate the quality of information provided by the community. The cbos are acutely aware of this issue and are highly motivated to provide the best possible information. Of the 13 national organizations interviewed, all have their material vetted by medical experts, and they train staff by drawing on the same up-to-date databases used by the medical system.

#### 3.1.2 Negotiation

Negotiating the medical system includes preparing for medical visits, advocating for oneself when necessary, and drawing on other services offered by the medical system such as nutrition, psychology, and rehabilatory medicine, among many others. Patients and family members are not passive recipients of physical care; their understanding of and participation in that care clearly influence the quality of care received. And benefits of physical health yet this fact remains obscure both to many professionals and to the lay public. Effective negotiation of the medical system requires that patients and their families not only assimilate information about their situation, but also draw on a skill set and attitude about their roles in care.

The cbos draw on the extensive experience of their members and play a critical role in teaching essential negotiating skills in a variety of ways, including, importantly, support groups. The power of support groups, beyond emotional and psychological support, lies in the sharing, by people with similar problems, of specific advice and practical solutions. Attendees learn about symptoms, medical care options, and available resources. The cbos arrange for people with the same tumour type (or other characteristics) to interact through support groups, information days, online resources, buddy systems, and similar mechanisms. This sharing of experience allows people to develop effective strategies in coping with treatment-related problems, to draw on the appropriate resources, and to demystify the medical experience. The cbos can provide an honest assessment of the medical system’s ability to provide care, outlining both its strengths and its weaknesses—an opinion that members of the medical system are reluctant to provide for various reasons.

#### 3.1.3 Physical Care: Surgery, Systemic Therapy, and Radiotherapy

Physical care is the only element of complete care in which the cbos have no direct role. However, anecdotally, it is possible for cbos to have some influence over the types of care being provided. They can help lobby governments to provide access to certain types of care (usually expensive). They can act as knowledge brokers to disseminate up-to-date professionally-created guidelines on the medical management of tumours. The provision of this information may be particularly helpful for rare tumours or in situations in which recent medical breakthroughs have not been widely disseminated. For instance, the standard of care for thyroid cancer has evolved over the years, and so there is likely a lag between recent developments in the standard of care and uptake by professionals. Regardless of whether gaps are present in the quality of care in Canada, patients can feel more reassured if they believe that they are exploring all their treatment options with information provided by a party outside of the medical system.

#### 3.1.4 Physical Health Promotion

The scientific literature has begun to show that exercise, a healthy diet, maintenance of a reasonable weight, sleep, and relaxation techniques can, in some cases, influence cancer outcomes^2^–^4^.

This information is reaching members of the medical community, who are increasingly educating their patients about the benefits of physical health promotion. But informing people about what is best for them is less effective than developing and delivering an integrated health promotion program run by highly trained staff. The latter service is rarely delivered in the Canadian medical system, perhaps with the exception of some breast cancer support programs. This is an unfortunate situation, because people who develop an active coping strategy often have a better quality of life than do those who take a passive approach.

The cbos are highly attuned to the patient perspective and want to help people focus their energies in helpful ways. Beyond promoting the message through multiple media, cbos can provide the actual programming. Whether by developing partnerships with university or community exercise specialists, arranging cooking classes, or teaching relaxation techniques, the cbos vastly increase capacity to deliver effective health promotion programs. The more than 1 million current Canadian cancer survivors themselves provide significant source of volunteers who can help to organize and lead the newly diagnosed to a healthier life.

#### 3.1.5 Psychological Care

Supporting cancer patients and families at the emotional and psychological level is a multifaceted task and not separable from all the other components of complete cancer care outlined in this article.

Psychological care can be categorized by the distress level of the person in need. The people who are most distressed—the 1 in 3 patients who suffer clinical depression or debilitating anxiety at some point in their cancer journey—need to be referred to a trained professional^5^.

Those suffering less distress can benefit from professionally-led psycho-educational support groups. People can also learn specific self-help skills for coping with difficult emotions and other stress-reduction techniques. Programs of this kind are offered by the medical system to some degree. Unfortunately, the budget for psychosocial care in Canada is very small: on average about 3% of the operating budget^6^.

Psychosocial professionals are often so busy taking care of the most distressed clients that they have very limited time to lead other programs, despite the fact that providing psychological care has been found to be a cost-saving initiative in dealing with a variety of other health problems such as arthritis, chronic pain, and heart disease. According to researchers Simpson *et al.*^7^, women in Alberta with breast cancer who participated in a psychological treatment group billed an average of $221 less over 2 years than did women in control conditions,

Regardless of the level of distress, psychological care should be available at every interaction with people affected by cancer. Cancer can be an extremely stressful and often isolating experience. Most people welcome the opportunity to discuss their experience, and studies show that simply listening to the patient and family members has a therapeutic effect^8^.

At a societal level, there is a need to normalize the emotional experience for the newly diagnosed. Medical professionals have a limited capacity to provide this basic form of psychological care because they are often under time constraints and may view their primary role as delivering physical care. Furthermore, the medical system appears appropriately reluctant to ask existing patients to volunteer as “buddies” for newly diagnosed patients. Issues of patient confidentiality and not wanting to burden patients in any way likely lie at the heart of this concern.

But, in the realm of providing psychological care, cbos are indispensable. Patients and families can quickly make contact with others who have travelled a similar journey. The cbos tap into a large network of survivors motivated to give back to their communities. These networks are especially helpful in rare tumours and in underserviced populations such as young adults. Evolving technologies such as chat rooms and the Web site at wikiCancer (www.wiki-cancer.org), where people can post their own stories and make themselves available to others with similar problems, mitigate against feelings of isolation in the newly diagnosed.

Furthermore, cbo staff and volunteers, often cancer survivors themselves, make time to listen to each and every person’s story. Often patients will describe an instant bond with someone who really “gets it”—a role that professionals, let alone family members, can never fulfil. Most patients feel stressed visiting cancer centres, and so having programs available in the comforting environments provided by cbos is also beneficial As with other elements of cancer care, the capacity to provide psychological programming is markedly increased with the involvement of the cbos.

#### 3.1.6 Meaning and Purpose

Many people will see the cancer diagnosis as an opportunity for personal growth. Others will find purpose in volunteering, fundraising, or supporting the recently diagnosed. Providing a way for people to find purpose in their cancer experience is therapeutic and a pillar of complete cancer care.

The medical system provides only very limited opportunities for survivors to contribute energy, expertise, and experience beyond financial donations. The cbos are the catalysts that convert this innate human tendency to give into concrete programs adapted quickly to the needs of others. Fundraising events, information days, and chat rooms have benefits far beyond providing practical and emotional support to the newly diagnosed. They are, in fact, a therapy unto themselves for everyone involved. And for those who want to explore their spirituality, the cbos offer well-evaluated programs that help people to find peace on their cancer journey.

### 3.2 The Influence of CBOS in Canada

The current popularity of cbos in Canada is testament to the value of the services they provide. Various organization types serve the perceived (and evaluated) needs of their clientele. Although the largest organization, the Canadian Cancer Society (ccs) funds clinical trials and research, its role in providing psychosocial care is impressive. An estimated 65,000 people access the ccs Cancer Information Service (cis) every year, at no cost, by telephone (90% of all contacts) or e-mail (10%) 9. Assuming 1 new cancer case per inquiry would imply that an estimated 15%–18% of new cancer patients, or people close to them, contact the cis each year. The ccs Web site draws more than 11,000 visits daily—one third of those to the About Cancer section, and about 900 to the Support Services section. Each year, the ccs’s Cancer Connection service matches each of more than 7000 clients with a trained volunteer having a similar diagnosis. The evaluations of these programs show that more than 95% of clients are very satisfied or satisfied with these services 10. The extent of the programs provided by the ccs extends far beyond those services into each provincial program and the dozens of regions and unit offices throughout the country.

An example of a national organization that caters to an underserved population is Young Adult Cancer Canada, whose mission is to serve the young adult cancer community. Although marketed only to the national community, the program links this techno-savvy group with innovative programs through their Web site (www.youngadultcancer.ca, which receives 152,000 visits annually), outreach programs, and help line.

A growing number of cbos are facility-based programs. For instance, Wellspring is a network of wellness centres set in the community. Typically, residential properties are renovated to create a warm and welcoming environment away from hospitals and clinics. Beyond impressive information services and professionally-led support groups and counselling services, Wellspring offers more than 28 programs such as yoga and relaxation classes, thus fulfilling the expressed needs of their “members.” Each program and centre is rigorously evaluated by its clients, allowing Wellspring to evolve quickly and respond effectively to the more than 40,000 visits annually by patients and caregivers across the Wellspring network.

InspireHealth is a Vancouver-based organization that demonstrates a truly integrative approach to cancer care. Its uniqueness is found in the provision to physicians of provincial government funding to offer a complete set of integrative services, with coaching for people on how to choose complementary therapies such as Chinese medicine. Each year InspireHealth serves 600 new patients. Their web-site (www.inspirehealth.ca), which, among its many features, includes a compilation of the best scientific studies on complementary therapies receives more than 110,000 hits annually.

## 4. CONCLUSIONS: TIME FOR COLLABORATION

Dozens of organizations exist to provide free support to a diverse group of patients. The time has come to recognize the incredible contribution, both in quality and quantity of care provided by these cbos. It is no longer acceptable to expect that patients and family members should have to negotiate two different systems of care. The medical community and the cbos need to collaborate and to learn from one another. Every person coming into contact with the people affected by cancer should refer the most distressed patients to professionals, and medical practitioners should refer their patients and their patients’ families to the cbos. For their part, the cbos will need to continue to evaluate and modify their programs with a research-based rigour. Seamless, high-quality psychosocial care, informed by up-to-date research and guided by wisdom, is in everyone’s interest.

## Figures and Tables

**TABLE I:** Community-based organizations attending the inaugural psychosocial oncology partners meeting

Canadian Breast Cancer Network
Canadian Cancer Society
Colorectal Cancer Association of Canada
Lung Cancer Canada
Leukemia and Lymphoma Society of Canada
National Ovarian Cancer Association
Ovarian Cancer Canada
Wellspring
RealTime Cancer
Willow Breast Cancer Support Canada
Hope and Cope

**TABLE II:** Essential components of complete cancer care

Information and skills to negotiate the medical system
Physical care: surgery, systemic therapy, and radiotherapy
Physical health promotion
Psychological care
Spiritual care
